# Evaluating Treatment Plan Modifications from Surgeons’ Initial Recommendations to Multidisciplinary Tumor Board Consensus for Cancer Care in a Resource-Limited Setting

**DOI:** 10.3390/curroncol32060310

**Published:** 2025-05-28

**Authors:** Sajida Qureshi, Waqas Ahmad Abbasi, Hira Abdul Jalil, Raheel Ahmed, Mubashir Iqbal, Hanieya Saiyed, Hira Fatima Waseem, Najeeb Naimatullah, Syed Rashidul Amin, Muhammad Saeed Quraishy

**Affiliations:** 1 Dow Medical College, Dow University of Health Sciences, Karachi 74200, Pakistan; waqas.abbasi@duhs.edu.pk (W.A.A.); dr.hkhan9@gmail.com (H.A.J.); raheel.ahmed@duhs.edu.pk (R.A.); mubiqe@gmail.com (M.I.); hanieya.saiyed@gmail.com (H.S.); saeed.quraishy@duhs.edu.pk (M.S.Q.); 2 School of Public Health, Dow University of Health Sciences, Karachi 75300, Pakistan; hira.waseem@duhs.edu.pk; 3 Department of Medical Oncology, Sindh Institute of Urology and Transplantation, Karachi 74200, Pakistan; snniamatullah@gmail.com; 4 Department of Nuclear Medicine and Molecular Imaging, Sindh Institute of Urology and Transplantation, Karachi 74200, Pakistan; syed.amin@siut.org

**Keywords:** multidisciplinary tumor board, clinical oncology, surgical oncology, curative therapy, palliative care

## Abstract

Multidisciplinary tumor boards (MTBs) are essential for optimizing cancer care through collaborative decision-making. However, the concordance between initial surgeons’ recommendations and MTB outcomes, particularly in resource-limited settings, remains underexplored. This study evaluates the agreement between treatment plans proposed initially by surgeons and those finalized through MTB discussions conducted at the same stage of patient evaluation, with a focus on changes in treatment intent between curative and palliative care. A retrospective analysis of 216 patients discussed at bi-weekly MTB meetings between January 2021 and December 2023 at a tertiary care hospital was conducted. Statistical tests, including kappa statistics and concordance analysis were applied to assess the interrater agreement between surgeon-recommended and MTB-finalized decisions and to evaluate changes in treatment intent. A *p*-value < 0.05 was considered statistically significant. Strong concordance and significant perfect agreement were observed between curative versus palliative decisions of surgeons and MTBs, (Cohen’s kappa = 0.89, *p* < 0.001). MTB recommendations were added to the surgeons’ suggested plans in 38.4% (n = 83) of cases and replaced them entirely in 25.0% (n = 54) of cases. Shifts in treatment intent from curative to palliative or vice versa were infrequent (2.31%, n = 5), specifically in esophageal and stomach cancers. MTB decisions achieved a 100% implementation rate. This study underscores the critical role of MTBs in collaborative decision-making and their value as an essential tool for consistent, individualized, and evidence-based cancer care.

## 1. Introduction

Cancer remains one of the most significant public health challenges of the 21st century, accounting for nearly one in every six deaths globally. According to the latest GLOBOCAN report, there were nearly 20 million new cancer cases worldwide, with 9.82 million of these occurring in Asia. Similarly, out of the total 9.7 million cancer-related deaths globally, 5.4 million were reported in Asia alone, representing a substantial portion of the global burden [1]. As advancements in cancer management continue to unfold, tumor staging and stage-adjusted treatment protocols have evolved. In an era of tailored treatment approaches, various factors such as staging methodologies, neoadjuvant and adjuvant therapies, evolving surgical techniques, and palliative care options all contribute to the complexity of treatment planning. Deciding on the most appropriate strategy can be difficult for individual clinicians at times [2]. Furthermore, it is often unrealistic for a single provider to be fully informed about the vast array of specialized treatment options across different subspecialties. To ensure that patients receive the most comprehensive and well-informed care, cancer treatment decisions are ideally recommended within a multidisciplinary tumor board (MTB). These MTBs bring together specialists from all areas of cancer care, including surgeons, oncologists, radiation oncologists, radiologists, pathologists, and nurse cancer coordinators, to discuss cases and formulate consensus-driven treatment plans. This model has been proven to provide the most effective format for deciding evidence-based management strategies [3], with improved patient selection and increased overall survival rates across various cancer types [4,5,6,7]. Additionally, the individual role of surgeons in MTBs is notable yet not quantified. Surgeons hold a leadership position in interdisciplinary teams [8] and are often regarded as key decision-makers in tumor board discussions, primarily due to their direct interaction with the patients and close clinical relationship, which enables them to determine the best course of action [9].

Despite the proven benefits of MTB discussions in optimizing cancer treatment decisions, this collaborative approach is not yet fully integrated into clinical practice in many low- and middle-income countries of Asia, specifically Pakistan, due to limited resources, time constraints, and institutional challenges [10]. Additionally, data on the effectiveness of MTB meetings in such settings are scarce, particularly regarding whether the patient management decisions guided by the MTB as a whole, including surgeons, align with individual surgeons’ initial decisions.

This study retrospectively examines the treatment decisions made during MTB meetings for cancer patients at a public sector tertiary care hospital. Specifically, it evaluates how often and to what extent the treatment plans initially recommended by surgeons were either upheld, modified, or entirely revised following MTB discussions, with an additional focus on the change of degree of preference in disease management from curative to palliative care or vice versa.

## 2. Materials and Methods

### 2.1. Study Design and Setting

The study was conducted according to the guidelines of the Declaration of Helsinki and approved by the Institutional Review Board of DUHS (IRB-2781/DUHS/1149). A retrospective study design was employed, and a review was conducted on medical records and the bi-weekly MTB meeting final decisions for all patients discussed between January 2021 and December 2023 at Surgery Unit-I, Dr. Ruth K.M. Pfau Civil Hospital, Karachi, Pakistan, a general surgery unit with specialized expertise in upper gastrointestinal (GI) surgery. The MTB was composed of all general and upper GI surgeons from the unit, as well as medical and radiation oncologists, radiologists, and pathologists. Meetings were held on a fixed bi-weekly schedule to ensure consistent participation and systematic case review. Standardized assessment tools, including ECOG performance status for functional evaluation, institutional radiology protocols for imaging review, and routine consideration of histopathological and laboratory parameters were used. These criteria ensured objective, reproducible, and multidisciplinary evaluation for all cases discussed.

### 2.2. Sample Size and Inclusion and Exclusion Criteria

Patients whose medical records were available and discussed during the bi-weekly MTB meetings within the defined study period were eligible for inclusion. Patient information was systematically retrieved from proformas, and only records with complete data, specifically, both the initial surgeons’ recommendations and final MTB decisions, were considered. Records lacking essential information, such as histology, grade, or stage, were excluded from the analysis (n = 9). To ensure data accuracy, all information underwent quality control checks. A total of 216 patients were included in the final analysis.

### 2.3. Data Analysis

Data analysis was performed using SPSS version 26. Descriptive statistics were used to report frequencies and proportions for categorical data, while mean and standard deviation were used for continuous (normal) data. One-way analysis of variance was used for continuous variables, and the Chi-square test and Fisher’s exact test were used for categorical variables such as age, gender, region of cancer, categories of histology, grade of differentiation, pre-/post-neoadjuvant, T stage, nodal stage, metastasis, and clinical stage to compare differences between the final MTB decisions. A pie chart was created to illustrate the percentage distribution of final decisions, and a bar chart was used to display the frequency distribution of curative versus palliative decisions made by surgeons and MTBs. Additionally, cross-tabulation was used to compare curative versus palliative decisions according to the region of malignancy. Inter-rater agreement between the curative versus palliative decisions of surgeons and MTBs was assessed using Cohen’s kappa (κ) statistic, which adjusts for agreement occurring by chance. Kappa values were interpreted as follows: κ < 0.20 = poor agreement, 0.21–0.40 = fair agreement, 0.41–0.60 = moderate agreement, 0.61–0.80 = substantial agreement, and 0.81–1.00 = almost perfect agreement. In addition, the concordance was also calculated to assess the accuracy of agreement between the curative versus palliative decisions of surgeons and MTBs. The concordance statistics closer to 1 indicated strong concordance. A *p*-value of <0.05 was considered statistically significant.

## 3. Results

### 3.1. Patient Demographics, Tumor Characteristics, and Treatment Decision Outcomes

Out of 216 patients, the average age of the patients was 47.73 ± 13.51 years, with 50.5% (n = 109) males and 49.5% (n = 107) females. Among all patients presented to the MTB panel, the most common malignancy was esophageal cancer (53.2%, n = 115), followed by colorectal, 14.4% (n = 31), stomach, 10.6% (n = 23), pancreatic, 9.3% (n = 20), breast, 7.4% (n = 16), abdominal wall, 1.9% (n = 4), retroperitoneal, 1.4% (n = 3), GE junction, 0.9% (n = 2), and others, 0.9% (n = 2).

**Figure 1:**
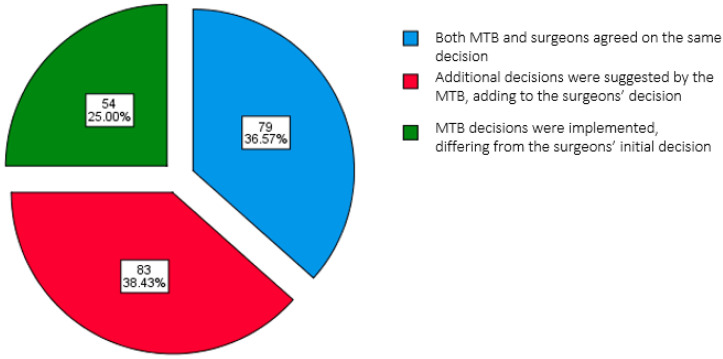
Pie chart showing the distribution of final decisions.

Among the cases, 63.0% (n = 136) had not received neoadjuvant therapy. Tumor staging (T stage) revealed that 2.4% (n = 5), 5.3% (n = 11), 13.6% (n = 28), 46.2% (n = 95), and 32.5% (n = 67) were classified as T0, T1, T2, T3, and T4, respectively. Lymph node involvement showed that 29.6% (n = 61) were N0, 51.9% (n = 107) were N1, 15.6% (n = 32) were N2, and 2.6% (n = 6) were N3. Metastasis was identified in 9.7% (n = 19) of cases. In terms of clinical staging, stage III was most prevalent (40.7%, n = 88), followed by stage IV (35.2%, n = 76), with stage II comprising 13.0% (n = 28) and stage I comprising 5.1% (n = 11) of the cases, while 6.0% (n = 13) remained unspecified. No significant associations were found between clinicopathological parameters, i.e., neoadjuvant therapy status, T stage, lymph node status, metastasis, or clinical stage, and final decision outcomes when checked for consensus between surgeons’ and MTB decisions (*p* > 0.05). Multidisciplinary decisions were based on a holistic clinical review for each case. These patient characteristics and their relationships with final decision outcomes are summarized in Table 1. Moreover, Figure 1 illustrates the distribution of final decisions implemented, where supplementary decisions were suggested by the MTB and added to the surgeons’ decision (38.4%, n = 83), both the MTB and surgeons agreed on the same decision (36.6%, n = 79), and MTB decisions that differed from the surgeons’ initial decision were implemented. These differences were either minor (changes in management strategy/plan while maintaining the same treatment intent) or major (changes in treatment intent between curative and palliative care) (25.0%, n = 54). Among these 54 cases, the primary factors influencing MTB decision changes included radiological staging (46.3%, n = 25), surgical fitness (22.2%, n = 12), histological findings (16.7%, n = 9), and clinical assessment parameters (14.8%, n = 8).

### 3.2. Agreement and Shifts in Curative vs. Palliative Decisions Between Surgeons and the MTB

**Figure 2:**
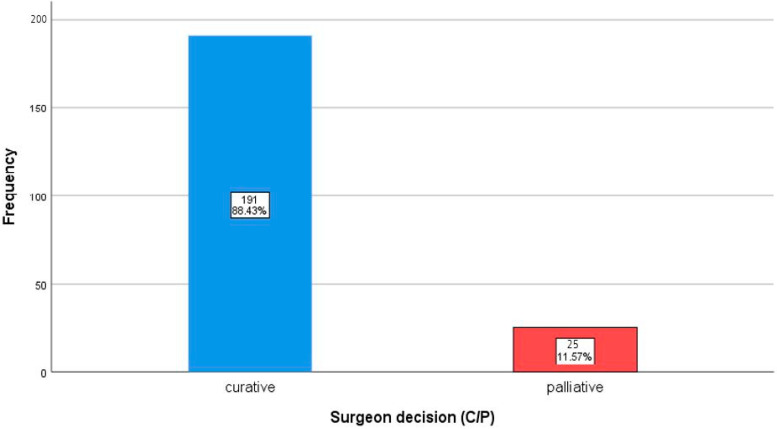

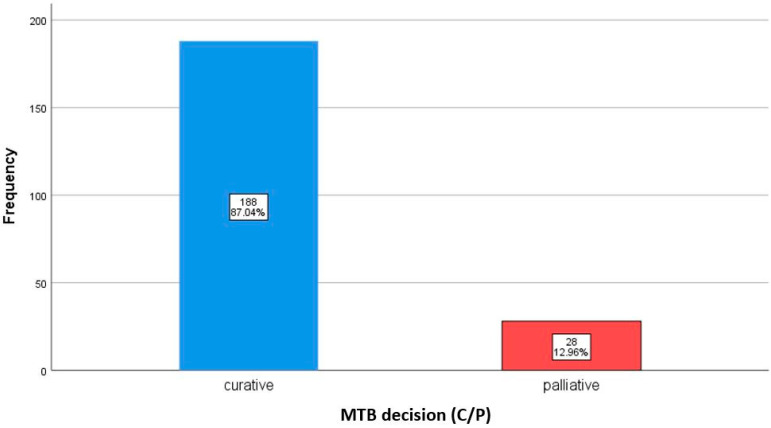
Bar chart showing the frequency distribution of curative vs. palliative decisions of **surgeons** and the MTB.

In analyzing the treatment intent proposed by both the surgeons and the MTB, a notable distribution between curative and palliative recommendations was observed. Initially, surgeons proposed curative treatment plans in the majority of cases, with 88.4% (191/216) designated as curative and 11.6% (25/216) as palliative. When reviewed by the MTB, these proportions shifted slightly, with 87.0% (188/216) of cases retaining a curative approach and 13.0% (28/216) adjusted to palliative intent. The distribution of curative versus palliative decisions made by surgeons and the MTB is shown in Figure 2.

Notably, among all 216 patients/cases, both the MTB and surgeons agreed on the same decision (curative, 99.5% [n = 187] and palliative, 85.7% [n = 24]), and MTB decisions differing from the surgeons’ decision were implemented (curative, 0.5% [n = 1] and palliative, 14.3% [n = 4]). The interrater agreement showed that there was a significant perfect agreement between the curative versus palliative decisions of surgeons and the MTB (Cohen’s kappa = 0.89, *p* < 0.001), and the concordance value of 0.97 showed strong concordance between the curative versus palliative decisions of surgeons and the MTB (Table 2).

To further understand these differences, MTB decisions were compared to the surgeons’ decisions, while taking into account the region of malignancy, as well as changes in treatment from “curative intent” to “palliative”. The MTB decisions aligned with the surgeons’ decisions in all regions of malignancy except for the esophagus, where the curative surgeons’ decision was altered to a palliative decision by the MTB in 14.3% (n = 2/14) of cases, and the stomach, where 16.7% (n = 2/7) of curative decisions were altered to palliative, while one palliative 6.7% (n = 1/16) decision was shifted to curative. In colorectal and breast regions, there was a strong concordance and significant perfect agreement between the curative versus palliative decisions of surgeons and the MTB (concordance = 1.00, Cohen’s kappa = 1.00, *p* < 0.001). Furthermore, strong concordance and significant perfect agreement were found between the curative versus palliative decisions of surgeons and the MTB in the esophagus (concordance = 0.98, Cohen’s kappa = 0.91, *p* < 0.001), while in the stomach region, there was substantial concordance and significant moderate agreement between the curative versus palliative decisions of surgeons and the MTB (concordance = 0.86, Cohen’s kappa = 0.68, *p* = 0.001) (Table 3). A detailed comparison of the curative versus palliative decisions of surgeons and the MTB according to region of malignancy is summarized in Table 3.

## 4. Discussion

This study provides an all-inclusive assessment of the concordance between initial surgeon-recommended treatment plans and collaborative MTB recommendations. While a moderate agreement was observed (36.6%, n = 79), slight discrepancies emerged in cases where additional input was suggested by the MTB, where addition refers to a complementary therapy, such as neoadjuvant chemotherapy before surgery, instead of upfront surgery. There were several other combinations of such additions, one of which is stated as an example, but these were not considerably detailed, as they were not included in our scope and it would have been technically difficult to deal with numerous decision combinations in population datasets as large as ours (n = 216). Furthermore, the MTB agreed on the same decision, as suggested initially by surgeons (38.4%, n = 83), and there were cases where the MTB’s decisions replaced the initial surgeons’ plan entirely (25.0%, n = 54), suggesting a radical change in the treatment strategy where decisions were often influenced by differing interpretations of radiological staging, histopathology, surgical fitness, or overall clinical assessment. These changes ranged from adjustments within the same treatment intent to major shifts between curative and palliative approaches, as reflected in Table 2. Such changes influenced by the MTB helped avoid unnecessary surgical interventions in patients with poor fitness, escalated treatment for those with more advanced disease on re-staging, and provided better personalized planning based on revised histology or imaging findings, illustrating the qualitative value of MTB consensus in aligning treatment with clinical appropriateness.

Notably, treatment intent shifted after MTB recommendations between curative and palliative in a small subset of cases, i.e., a major change (2.31%, n = 5), primarily in esophageal and stomach malignancies. These differences likely reflect the inherent complexity of tumor characteristics in the gastrointestinal (GI) region, which complicates clinical decision-making.

Importantly, with GI malignancies comprising a major cancer burden in Asia [11,12], the predominance of upper GI cases in our cohort is aligned with national trends. Esophageal cancer ranks among the top five cancers in Pakistan (GLOBOCAN 2022), and regional data from the Karachi Cancer Registry (2017–2021) confirm similar patterns. Our general surgery unit, recognized for upper GI surgical expertise, serves as a referral hub for complex cases, contributing to the high proportion of GI, specifically esophageal malignancies reviewed by MTBs [1,13].

Our findings are also consistent with those of Hagen et al., who reported that 34.5% (87/252) of initial management plans proposed by surgeons were modified following MTB discussions, with 6.4% (16/252) involving a shift from curative to palliative treatment intent [14]. In our cohort, MTB recommendations led to a complete change in the surgeons’ plans in 25.0% (n = 54) of cases, which is lower. Additionally, shifts in treatment intent were rare, occurring in only 5 out of 216 cases. This low frequency underscores the precision of surgeons’ initial assessments, establishing surgeons as key contributors and leaders within multidisciplinary teams [8].

Recent studies across various malignancies have demonstrated the significant impact of MTB discussions on treatment planning. For instance, changes in treatment plans post-MTB discussions were observed in 66% of breast cancer cases, with an 83.7% implementation rate [15]. Similarly, 72.2% of pancreatic lesion plans were revised [16], 31% of colorectal cancer decisions were modified [17], and 22% of head and neck cancer recommendations were altered [18] as a result of MTB meetings. In our cohort, MTB discussions led to modifications in the initial treatment plans in a substantial proportion of cases (25%), with a 100% implementation rate.

Similarly, previous studies have consistently highlighted the long-term benefits of MTB discussions in optimizing treatment outcomes. Basendowah et al. demonstrated that MTB involvement significantly reduced overall mortality in patients with mixed GI cancers and notably improved survival in stomach cancer [19]. Freytag et al. also reported that patients participating in three or more MTB meetings had significantly improved overall survival, underscoring the long-term value of regular MTB review [20]. These findings align with similar evidence across various malignancies, including gynecological, breast, urological, and head and neck cancers, underscoring the essential role of MTBs in enhancing patient care [21]. While our study did not assess long-term outcomes due to its defined scope, we observed that most MTB recommendations resulted in additions to existing surgical plans, with only a few leading to major alterations. This may reflect the strength of initial clinical assessments, particularly in complex cases where experienced surgeons often develop robust management strategies early on.

Interestingly, no significant associations were found between clinicopathological characteristics (e.g., tumor stage, grade, metastasis) and the concordance between surgeons’ and MTB decisions, as detailed in Table 1. This may suggest that the decision differences were not solely driven by measurable disease parameters, but rather by broader clinical insights discussed during MTB evaluations, such as performance status, imaging nuances, or other factors that are not included in our analysis. Moreover, the inclusion of diverse cancer types and variations in the timing and progression stages of cases upon presentation to MTBs could be another factor in not finding a significant correlation with the decisions in Table 1, as far as the uniformity of the dataset is concerned. This is due to the urgency of obtaining collaborative advice in complex cases upon presentation. Notably, over 50% of cases in our cohort were advanced-stage (stage III and IV), contributing to their complexity [22].

Evidence on the use of MTBs in low- and middle-income countries (LMICs) is still limited compared to that in high-income countries (HICs). In LMICs, MTBs often face challenges such as limited diagnostic tools, shortage of specialists, and weak healthcare infrastructure [23]. However, similar to our findings, available studies show that MTBs can still play a valuable role. In Rwanda, MTBs contributed to treatment planning in 85% of cases, showing they can support clinical decisions even with resource constraints [24]. In Uganda, 57% of the 226 management decisions made by MTBs were implemented, reflecting both their impact and the general overall need for better systems to follow through [25].

Additionally, in a study of 1421 patients, Lamb et al. [26] showed that incomplete diagnostic data and poor referrals hinder MTB decisions. Our study already addressed these limitations by excluding cases with missing radiological, pathological, or referral data, as stated in our methodology, ensuring that only patients with complete clinical information were included. This allowed for a clearer understanding of MTB influence on decision-making, free from confounding information gaps.

This study was conducted at a single tertiary care center and employed a retrospective design, which may limit generalizability. Additionally, while our analysis focused on concordance and shifts in treatment recommendations, long-term outcomes such as recurrence and survival were beyond the study’s scope. Future prospective, multi-center studies evaluating the impact of MTB-influenced decisions on patient outcomes are warranted to validate and extend these findings.

Despite these limitations, our study highlights the important role of MTBs in refining treatment strategies, especially in resource-limited settings. It underscores the need for structured, collaborative decision-making to ensure individualized, evidence-based cancer care.

## Figures and Tables

**Table 1:** Distribution of patients’ characteristics according to final decision (n = 216).

		Final Decision				
		Both the MTB and Surgeons Agreed on the Same Decision	Additional Decisions Were Suggested by the MTB, Adding to the Surgeons’ Decision	MTB Decisions Differing from the Initial Surgeons’ Decision Were Implemented	Total	*p*-Value
		n = 79 (%)	n = 83 (%)	n = 54 (%)	n = 216(%)	
Age, Mean ± SD (years)		48.14 ± 14.15	48.17 ± 13.53	46.44 ± 12.62	47.73 ± 13.51	0.725
**Gender**						
	Male	36 (45.6)	45 (54.2)	28 (51.9)	109 (50.5)	0.531
	Female	53 (54.4)	36 (45.8)	26 (48.1)	107 (49.5)	
**Region of Cancer**						
	Esophagus	48 (60.8)	41 (49.4)	26 (48.1)	115 (53.2)	N/A
	Colorectal	10 (12.6)	14 (16.9)	7 (13.0)	31 (14.4)	
	Stomach	4 (5.1)	8 (9.6)	11 (20.3)	23 (10.6)	
	Pancreas	14 (17.6)	3 (3.8)	3 (5.6)	20 (9.3)	
	Breast	1 (1.3)	9 (10.8)	6 (11.1)	16 (7.4)	
	Abdominal wall	1 (1.3)	3 (3.6)	0 (0)	4 (1.9)	
	Retroperitoneal	0 (0)	3 (3.5)	0 (0)	3 (1.4)	
	GE junction	1 (1.3)	1 (1.2)	0 (0)	2 (0.9)	
	Others	0 (0)	1 (1.2)	1 (1.9)	2 (0.9)	
**Categories of Histology**						
	Carcinoma of esophagus	50 (63.3)	45 (54.2)	26 (48.1)	121 (56.0)	N/A
	Colorectal adenocarcinoma	8 (10.1)	10 (12.0)	7 (13.0)	25 (11.6)	
	Adenocarcinoma of stomach	3 (3.8)	8 (9.6)	11 (20.4)	22 (10.2)	
	Carcinoma of breast	1 (1.3)	9 (10.8)	5 (9.3)	15 (6.9)	
	Periampullary adenocarcinoma	11 (13.9)	2 (2.4)	2 (3.7)	15 (6.9)	
	Abdominal wall tumors	1 (1.3)	3 (3.6)	0 (0)	4 (1.9)	
	Pancreatic tumors	4 (5.1)	0 (0)	0 (0)	4 (1.9)	
	Tetroperitoneal tumors	0 (0)	2 (2.4)	0 (0)	2 (0.9)	
	Phyllodes tumor of breast	0 (0)	0 (0)	2 (3.7)	2 (0.9)	
	Pheochromocytoma	0 (0)	1 (1.2)	0 (0)	1 (0.5)	
	Cholangiocarcinoma	0 (0)	0 (0)	1 (1.9)	1 (0.5)	
**Grade of Differentiation**						
	Well	20 (25.3)	13 (15.7)	12 (22.2)	45 (20.8)	0.072
	Moderate	43 (54.4)	46 (55.4)	20 (37.0)	109 (50.5)	
	Poor	7 (8.9)	16 (19.3)	15 (27.8)	38 (17.6)	
	Not specified	9 (11.4)	8 (9.6)	7 (13.0)	24 (11.1)	
**Pre-/Post-Neoadjuvant**						
	Pre	47 (59.5)	55 (66.3)	34 (63.0)	136 (63.0)	0.457 ^
	Post	32 (40.5)	26 (31.3)	20 (37.0)	78 (36.1)	
	Not specified	0 (0)	2 (2.4)	0 (0)	2 (0.9)	
**T Stage (n = 206)**						
	T0	2 (2.7)	2 (2.5)	1 (1.9)	5 (2.4)	0.997 ^
	T1	5 (6.7)	4 (5.1)	2 (3.8)	11 (5.3)	
	T2	12 (16.0)	10 (12.7)	6 (11.5)	28 (13.6)	
	T3	33 (44.0)	37 (46.8)	25 (48.1)	95 (46.2)	
	T4	23 (30.7)	26 (32.9)	18 (34.6)	67 (32.5)	
**Node Stage (n = 206)**						
	N0	23 (30.7)	22 (27.8)	16 (30.8)	61 (29.6)	0.894 ^
	N1	38 (50.7)	44 (55.7)	25 (48.1)	107 (51.9)	
	N2	13 (17.3)	10 (12.7)	9 (17.3)	32 (15.6)	
	N3	1 (1.3)	3 (3.8)	2 (3.8)	6 (2.9)	
**M: Metastasis (n = 198)**						
	MO	64 (91.4)	66 (85.7)	47 (95.9)	177 (90.3)	0.169 ^
	M1	6 (8.6)	11 (14.3)	2 (4.1)	19 (9.7)	
**Clinical Stage**						
	Stage I	27 (34.2)	30 (36.1)	19 (35.2)	11 (5.1)	0.754 ^
	Stage II	6 (7.6)	3 (3.6)	2 (3.7)	28 (13.0)	
	Stage III	8 (10.1)	15 (18.1)	5 (9.3)	88 (40.7)	
	Stage IV	32 (40.5)	31 (37.3)	25 (46.3)	76 (35.2)	
	Not specified	6 (7.6)	4 (4.8)	3 (5.6)	13 (6.0)	

N/A means not applicable, and the *p*-value was calculated by using the chi-square and ^ Fisher’s exact tests for categorical variables and one-way ANOVA for continuous (age) variable.

**Table 2:** Distribution of curative versus palliative decisions of surgeons and the MTB (n = 216).

		MTB Decision		Concordance	Cohen’s Kappa	*p*-Value
		Curative (n = 188)	Palliative (n = 28)			
**Surgeons’ Decision**	Curative (n = 191)	187 (99.5)	4 (14.3)	0.97	0.89	<0.001 *
	Palliative (n = 25)	1 (0.5)	24 (85.7)			

* The *p*-value was calculated by using Cohen’s kappa.

**Table 3:** Comparison of the curative versus palliative decisions of surgeons and the MTB according to region of malignancy.

Region of Malignancy	Surgeons’ Decision	MTB Decision		Concordance	Cohen’s Kappa	*p*-Value
		n (%)	n (%)			
**Colorectal**		**Curative (n = 27)**	**Palliative** **(n = 4)**			
	**Curative (n = 27)**	27 (100.0)	0 (0)	1.00	1.00	<0.001 *
	**Palliative (n = 4)**	0 (0)	4 (100.0)			
**Breast**		**Curative (n = 15)**	**Palliative (n = 1)**			
	**Curative (n = 15)**	15 (100.0)	0 (0)	1.00	1.00	<0.001 *
	**Palliative (n = 1)**	0 (0)	1 (100.0)			
**Esophagus**		**Curative (n = 101)**	**Palliative (n = 14)**			
	**Curative (n = 103)**	101 (100.0)	2 (14.3)	0.98	0.91	<0.001 *
	**Palliative (n = 12)**	0 (0)	12 (85.7)			
**GE Junction**		**Curative (n = 1)**	**Palliative (n = 1)**			
	**Curative (n = 1)**	1 (100.0)	0 (0)	1.00	1.00	0.157
	**Palliative (n = 1)**	0 (0)	1 (100.0)			
**Stomach**		**Curative (n = 16)**	**Palliative (n = 7)**			
	**Curative (n = 17)**	15 (93.3)	2 (16.7)	0.86	0.68	0.001 *
	**Palliative (n = 6)**	1 (6.7)	5 (83.3)			
**Others**		**Curative (n = 1)**	**Palliative (n = 1)**			
	**Curative (n = 1)**	1 (100.0)	0 (0)	1.00	1.00	0.157
	**Palliative (n = 1)**	0 (0)	1 (100.0)			
**Pancreas**		**Curative (n = 20)**	**Palliative (n = 0)**			
	**Curative (n = 20)**	20 (100.0)	0 (0)	N/A	N/A	N/A
**Retroperitoneum**		**Curative (n = 3)**	**Palliative (n = 0)**			
	**Curative (n = 3)**	3 (100.0)	0 (0)	N/A	N/A	N/A
**Abdominal Wall**		**Curative (n = 4)**	**Palliative (n = 0)**			
	**Curative (n = 4)**	4 (100.0)	0 (0)	N/A	N/A	N/A

N/A means not applicable, and the *p*-value was calculated by using Cohen’s kappa; (*) represents significance.

## Data Availability

The data presented in this study are available from the corresponding author on request. The data are not publicly available due to ethical restrictions.
